# Clinical characteristics, imaging findings, management, and outcomes of patients with scimitar syndrome at a tertiary referral healthcare center in Colombia

**DOI:** 10.1007/s10554-024-03102-1

**Published:** 2024-04-18

**Authors:** Ana M. Aristizabal, Carlos A. Guzmán-Serrano, Nancy Vanessa Mondol-Villamil, Lina Maria Bolaños-Vallejo, Valentina Mejia-Quiñones, Maria Alejandra Recio-Gómez, Enrique Carlos García-Pretelt, Mauricio Mejía-González, Walter Mosquera Alvarez, Jaiber Alberto Gutiérrez-Gil

**Affiliations:** 1 Facultad de Ciencias de la Salud, Facultad de Ciencias de la Salud, Universidad Icesi, Cali, Colombia; 2 Departamento materno-infantil, Fundación Valle del Lili, Cardiología pediátrica, Cali, Colombia; 3 Centro de Investigaciones Clínicas, Fundación Valle del Lili, Av. Simón Bolívar - Carrera 98 # 18-49 Cali, Cali, Colombia; 4 Departamento de Radiología e imágenes Diagnósticas, Fundación Valle del Lili, Cali, Colombia

**Keywords:** Scimitar syndrome, Chest radiography, Computed tomography, Magnetic resonance, Echocardiography, Heart disease

## Abstract

Scimitar Syndrome is part of a complex spectrum of congenital cardiovascular anomalies related to anomalous pulmonary venous return. Depending on the extent of involvement, treatment can be either expectant or surgical. Prognosis and survival have been controversial, with some results supporting early surgical management. This research aims to disclose the outcomes and describe the management, clinical and imaging characteristics of patients diagnosed with Scimitar Syndrome treated in a tertiary referral healthcare center. Longitudinal descriptive observational study. The study included all patients diagnosed with scimitar syndrome in our institution between January/2011 and December/2022. A description of the sociodemographic and clinical characteristics, diagnostic tools used, treatment features, and patient outcomes is provided. Eleven patients were included, with a mean age at diagnosis of five years (CI 0–17), six of which were female (54.55%). Nine (81.82%) patients had evidence of a scimitar vein on the chest radiograph, six (54.55%) cardiac dextroposition, six (54.55%) pulmonary hypoplasia, five (45.45%) right pulmonary artery hypoplasia, and three (27.27%) had aortopulmonary collaterals. Four (36.36%) patients had horseshoe lungs, and four (36.36%) had bronchopulmonary sequestration. In the associations, two (18.18%) patients were found to have an atrial septal defect, three (27.27%) ventricular septal defect, and one (9%) had Tetralogy of Fallot. Pulmonary hypertension was demonstrated in two (18.18%) patients. Seven (63.64%) required surgical management to correct the scimitar vein, and two patients died due to unrelated complications. Scimitar syndrome presents diagnostic and treatment challenges, necessitating a multidisciplinary approach for timely care. Chest radiography and CT scans are primary diagnostic tools, with surgical intervention often warranted alongside other heart defects or significant hemodynamic repercussions. Medical management is effective for mild to moderate cases. Long-term patient outcomes remain uncertain due to study limitations, but improved life expectancy is anticipated with ongoing care.

## Introduction

Scimitar syndrome, also known as hypogenetic lung, is named after the observation of a curved vascular structure resembling a Turkish scimitar sword at the base of the right lung [1]. It is part of a complex spectrum of congenital cardiovascular anomalies related to anomalous pulmonary venous return [1]. In this condition, there is an aberrant venous return from the pulmonary veins to the inferior vena cava, the cavoatrial junction, or the right atrium, which can be partial or complete depending on the number of affected pulmonary veins [1, 2]. It was first described in 1836 based on the autopsy of an infant with dextroposition of the heart and hypoplasia of the right lung. Subsequently, it was reported for the second time in 1949 in an asymptomatic living patient through cardiac catheterization [2]. The first reported surgical intervention took place in 1950 in a patient with recurrent pneumonia, and the first corrective surgery was performed in 1956 [2]. However, it wasn’t until 1960 when the syndrome was firstly named, characterized and associated to radiologic findings [3].

Its incidence is low, with an estimated prevalence of 1 to 3 per 100,000 births. Partial presentation in the right pulmonary veins is more common, although there are variations of the syndrome where their left counterparts are affected [2, 4]. Some of these patients will have other congenital defects such as hypoplasia of the right lung, dextroposition of the heart, anomalous arterial blood supply to the right lower lobe, and less frequently diaphragmatic defects or horseshoe lung, among others [4, 5]. Similarly, three-quarters of the patients may concurrently present associated heart malformations such as atrial or ventricular septal defects, aortic coarctation, or Tetralogy of Fallot [6–8].

Its diagnostic approach relies on clinical evaluation supported by diagnostic imaging, although there are no guidelines or criteria to decide which imaging technique is preferred over another. Diagnostic suspicion can be raised through chest radiography, where the “scimitar sign” might be observed, along with dextroposition, in addition to other findings such as the presence of atelectasis or pulmonary agenesis [9, 10]. Furthermore, three-dimensional computed tomography (CT) scan and cardiac magnetic resonance imaging (MRI) allow for the anatomical visualization of venous malformations, aiding in ruling out other associated conditions such as horseshoe lung, or when chest radiography is inconclusive but suspicion remains high [9, 10]. Also, the use of echocardiography is regarded as one of the primary imaging modalities in pediatric cardiology for identifying concurrent congenital heart conditions, and it can assist in outlining the scimitar vein [9]. Nevertheless, cardiac catheterization and angiography are ideal for establishing the hemodynamic conditions of the patient such as assessing the degree of left-to-right shunt and pulmonary hypertension, providing clearer visualization of pulmonary arterial and venous anatomy, identify systemic arterial collaterals from the thoracoabdominal aorta to the lung, and detecting other concurrent conditions [9]. Therefore, these latter methods are considered optimal for diagnosis, although they are not always required.

This study aims to describe the experience from a tertiary referral healthcare center in Colombia regarding clinical characteristics, diagnostic tools used, management given and the outcomes on follow-ups of patients who received surgical intervention, and those who did not, of the Scimitar syndrome.

## Methods

An observational descriptive study on a longitudinal cohort, this research involved retrospective data collection from patients between January 2011 and June 2022. Patient selection criteria for inclusion encompassed age, sex, comorbidities, and severity of symptoms or disease presentation. Patients with a non-specified or yet-to-be-proven diagnosis of Scimitar syndrome and those with unclear or incomplete information regarding variables were excluded.

The variables were categorized into five modules. The sociodemographic module retrieved information about birth date, diagnostic date, age at diagnosis, and sex. The clinical module evaluated the presence of unspecified symptoms, cyanosis, recurrence of pneumonia or respiratory tract infections, pulmonary hypertension, heart failure, and New York Heart Association (NYHA) class. The diagnostic module assessed whether the patient had diagnostic images such as chest radiography, chest CT scan, electrocardiogram, echocardiogram, and MRI. Considering that there are no international guidelines for this disease, the selection of each type of diagnostic imaging was based on the diagnostic suspicion of the physician, whether the findings were conclusive or not in previously taken images, whether patients had recent images taken institutionally or from another institution, and whether there was hemodynamic repercussion or not. The treatment module collected information about the necessity of surgical treatment, surgical times, the Risk Adjustment for Congenital Heart Surgery classification (RACHS- 2), Scimitar vein correction, and the necessity for ligation/embolization of the vascular supply to the sequestrated lobe. The monitoring variables evaluated various parameters including the duration of hospitalization, length of stay in the intensive care unit, duration of intubation, and mortality rates among surgical patients. Additionally, the presence of at least one postoperative follow-up session from the time of diagnosis or surgical intervention until the data analysis phase in June 2023 was taken into account for all participants. Special attention was given to identifying complications arising from surgical interventions in patients undergoing corrective procedures, as well as the emergence of supplementary symptoms leading to hospital admissions in cases of non-operated Scimitar syndrome patients.

A univariate analysis of quantitative variables was made using the Shapiro-Wilk test to assess their distribution. For normally distributed variables, the mean and standard deviation will be used, while non-normally distributed variables median and interquartile ranges were used. Qualitative variables will be presented in frequency tables as percentages. Outcomes will be expressed as a proportion, indicating successful outcomes in surgically treated Scimitar Syndrome patients over the total diagnosed cases.

A total of thirteen patients diagnosed with Scimitar syndrome, with or without associated lesions, who received medical or surgical treatment within the institution were reviewed. After evaluating the inclusion and exclusion criteria, a total of 11 patients were included in the study. The present study was reviewed and approved by the institutional ethics committee. Due to the retrospective nature of the protocol, it was approved without the need for informed consent.

## Results

A total of 11 patients were included, 5 (45.45%) males and 6 (54.55%) females, with a mean age of 5 years. Nine patients (81.82%) presented with symptoms, and 2 (18.18%) had a diagnosis of pulmonary hypertension. By history, 5 (45.45%) patients reported cyanosis, 4 (36.36%) recurrent respiratory infections, and 3 (27.27%) experienced recurrent pneumonias. Concerning functional capacity, 3 patients (27.27%) were in NYHA class I, five patients (45.45%) were in class II, and three patients (27.27%) were in class III (Table 1).

**Table 1:** Sociodemographic and clinical characteristics of the patients. *Mean

		*n* = 11 (%)
Age (Years)	4,64 (0–17)*	
Sex	Male	5(45,45%)
	Female	6(54,55%)
Symptomatic	Yes	9(81,82%)
	No	2(18,18%)
Clinical findings	Cyanosis	5(45,45%)
	Recurrent pneumonia	3(27,27%)
	Recurrent respiratory infections	4(36,36%)
	Pulmonary hypertension	2(18,18%)
NYHA functional class	Class I	3(27,27%)
	Class II	5(45,45%)
	Class III	3(27,27%)
	Class IV	0(0)

Regarding the cardiopulmonary anatomical component, five of the patients (45.45%) had dextroposition of the heart, 5 (45.45%) had hypoplasia of the right pulmonary artery, and 3 (27.27%) had aortopulmonary collateral arteries. Concomitantly, it was found that three patients (27.27%) had a congenital heart defect associated. Two patients (18.18%) had atrial septal defects, three (27.27%) had ventricular septal defects and one (9%) had Tetralogy of Fallot. Out of the total patients, 2 (18.18%) developed pulmonary hypertension detected on cardiac catheterization. The mean diameter of the left pulmonary artery was 12.94 mm, and the mean diameter of the right pulmonary artery was 9.88 mm, with a mean pulmonary artery diameter of 18.55 mm (Table 2) 1, 2, 3 and 4.

**Table 2:** Diagnostic characteristics of the patients. *Mean (SD)

		*n* = 11 (%)
***Chest radiography***	10 (90,91%)	
Presence of Scimitar vein	9 (81,82%)	
Cardiac dextroposition	6 (54,55%)	
***Chest computed tomography***	9 (81,82%)	
Presence of Scimitar vein	9 (81,82%)	
Scimitar vein drainage	Intrahepatic	3 (27,27%)
	Suprahepatic	6 (54,55%)
Pulmonary hypoplasia	6 (54,55%)	
Horseshoe lung	4 (36,36%)	
Cardiac dextroposition	5 (45,45%)	
Atresia or hypoplasia of the pulmonary artery	Right	5 (45,45%)
Aortopulmonary collateral arteries	3 (27,27%)	
Bronchopulmonary sequestration	4 (36,36%)	
Laterality of bronchopulmonary sequestration	Right	4 (36,36%)
Cardiac findings of pulmonary hypertension	5 (45,45%)	
Atrial septal defect	2 (18,18%)	
Ventricular septal defect	3 (27,27%)	
Left pulmonary artery diameter (mm)	12,94 (1,94)*	
Right pulmonary artery diameter (mm)	9,88 (1,46)*	
Pulmonary artery diameter (mm)	18,55 (2,23)*	
***Magnetic resonance***	2(18,18%)	
Presence of Scimitar vein	2 (18,18%)	
Laterality	Right	2 (18,18%)
Scimitar vein drainage	Intrahepatic	1 (9,09%)
	Suprahepatic	1 (9,09%)
Pulmonary hypoplasia	1 (9,09%)	
Atresia or hypoplasia of the pulmonary artery	Right	1 (9,09%)
Bronchopulmonary sequestration	1 (9,09%)	
Laterality of bronchopulmonary sequestration	Left	1 (9,09%)
	Right	1 (9,09%)

**Fig. 1:**
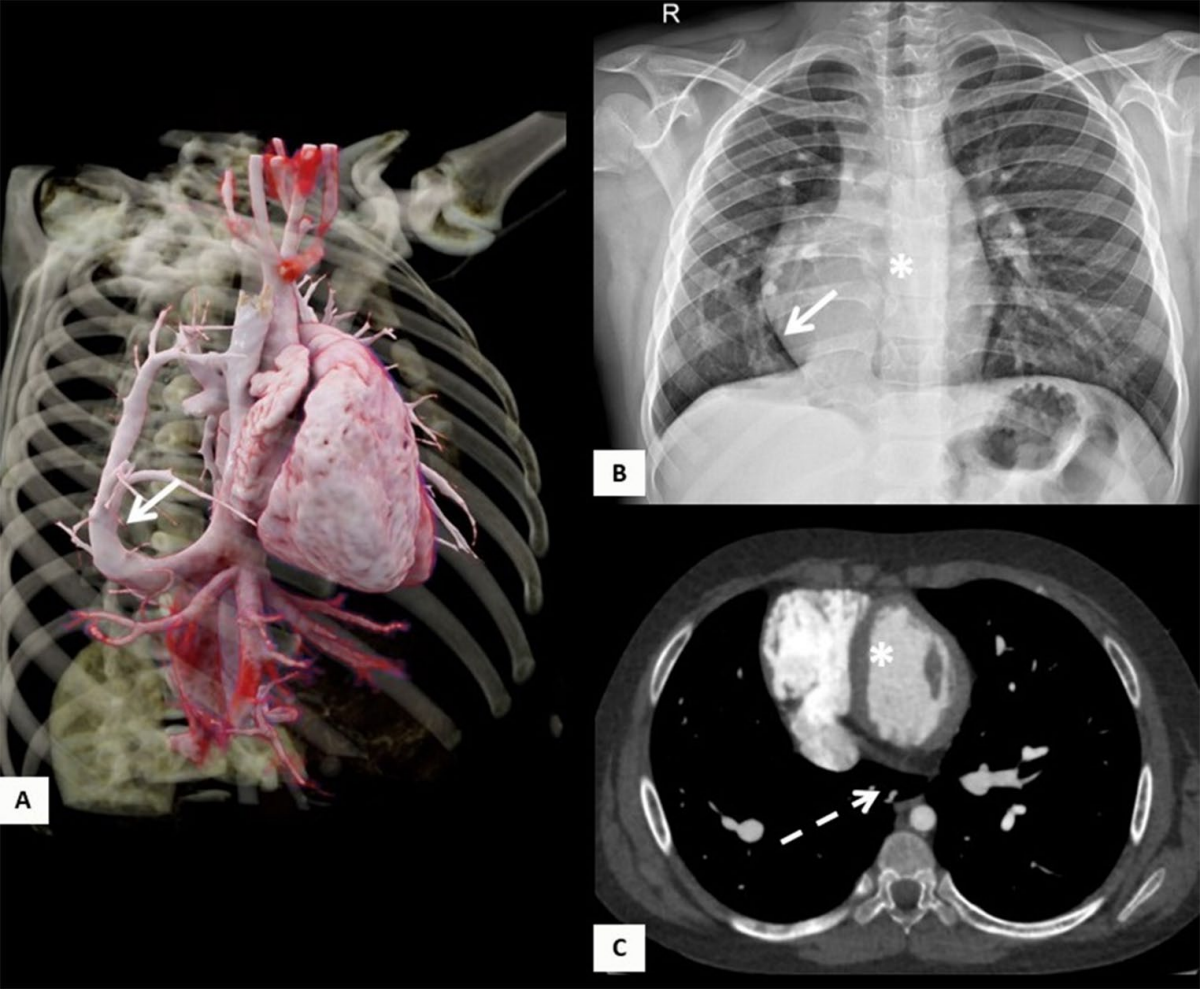
8-year-old male patient with Scimitar syndrome. (**A**) 3D rendered chest image and (**B**) chest X-ray, show anomalous right venous drainage into the suprahepatic inferior vena cava (white arrow). (**C**) Chest CT angiography shows mediastinal midline shift with dextroposition of the heart (asterisk), and horseshoe lung (dashed white arrow)

In terms of imaging, nine patients (81.82%) had evidence of a Scimitar vein on chest X-ray, and 6 (54.55%) had dextroposition of the heart. Nine patients (81.82%) had chest CT scans, which showed the presence of the Scimitar vein in the images; six (54.55%) of these patients had suprahepatic drainage of the Scimitar vein, and 3 (27.27%) had infrahepatic drainage. Pulmonary hypoplasia was reported in 6 patients (54.55%), and horseshoe lung in 4 (36.36%). Four (36.36%) of the patients had bronchopulmonary sequestration, all on the right side.

**Fig. 2:**
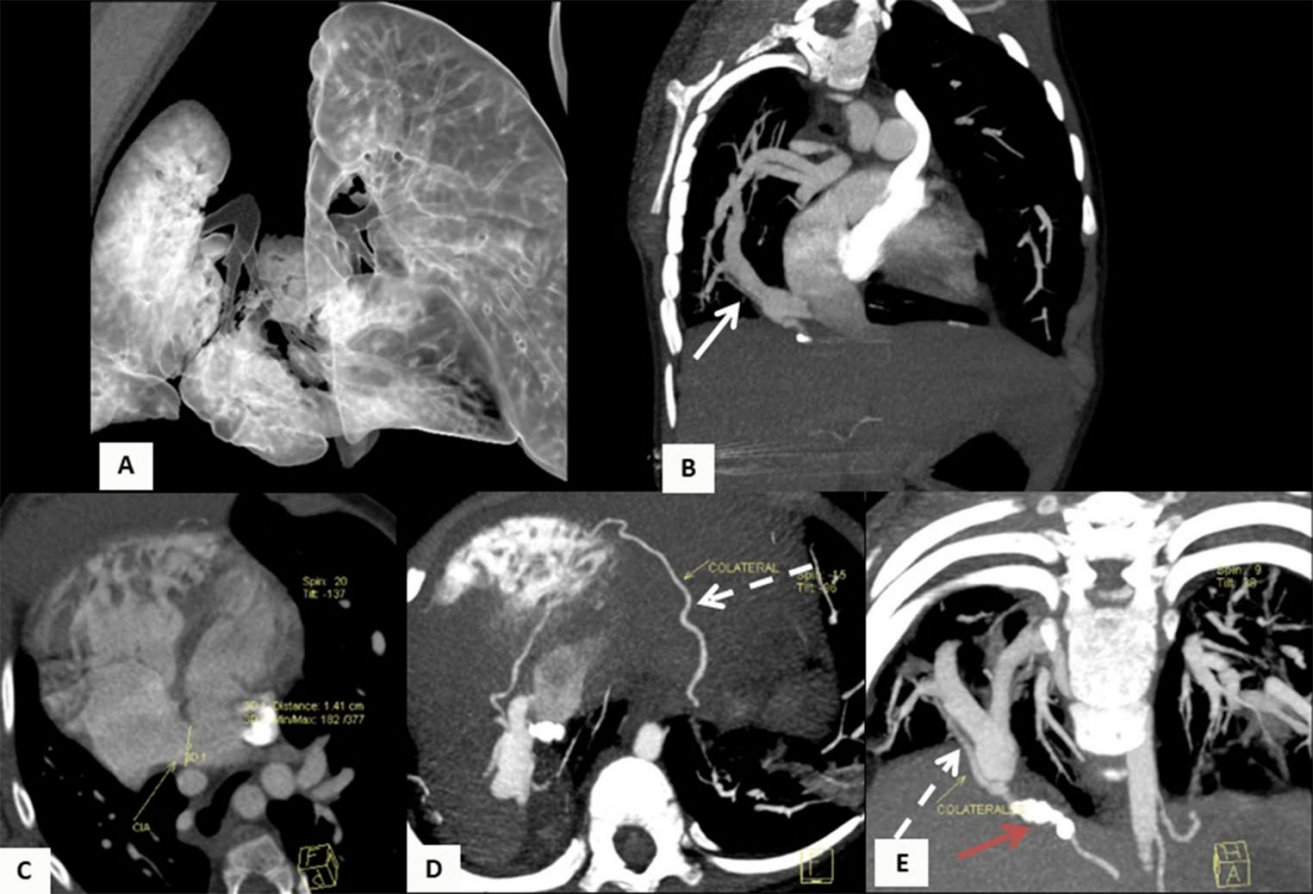
3-year-old male patient with Scimitar syndrome. (**A**) 3D rendered lungs image shows decreased volume in the right lung. (**B**-**E**) Chest Angio-CT with multiplanar reconstructions. (**B**) Maximum intensity projection (MIP) oblique-coronal reconstruction shows anomalous right pulmonary venous drainage into the inferior vena cava (white arrow). (**C**) Axial reconstruction shows a large atrial septal defect (ASD) as part of the Scimitar syndrome. (**D**) MIP oblique-axial maximum intensity projection and (**E**) MIP oblique-coronal reconstruction, show aortopulmonary collateral circulation (dashed arrows) arising from diaphragmatic arteries directed towards the connection point between the anomalous venous drainage and the inferior vena cava, with post-embolization changes (red arrow)

**Fig. 3:**
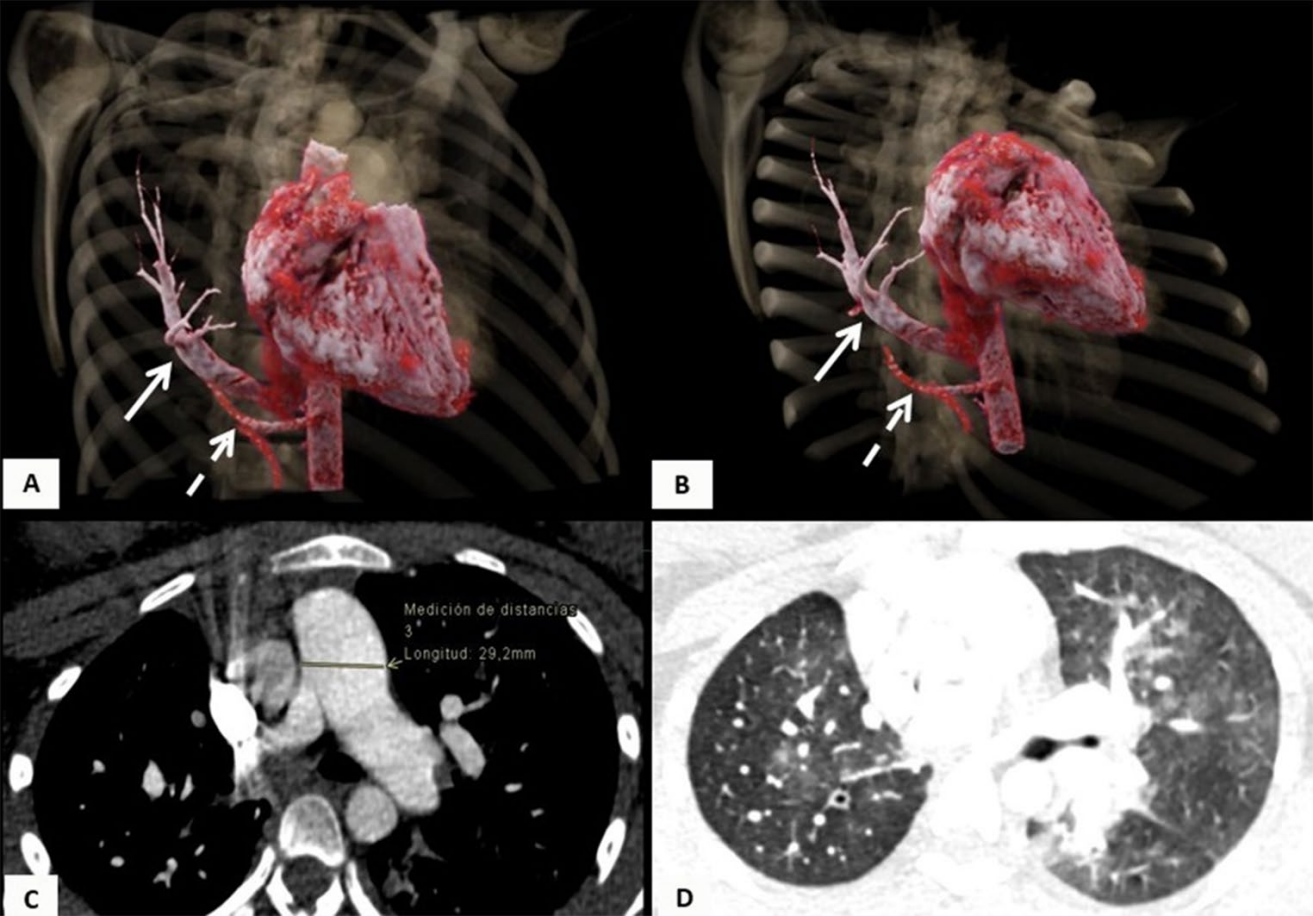
20-year-old female patient with Scimitar syndrome. (**A**-**B**) 3D rendered reconstructions of the heart and chest vessels depict anomalous right pulmonary venous drainage into the inferior vena cava (white arrow), also known as Scimitar vein, and an artery originating from the aorta supplying the right lower lobe (Dashed arrow), indicative of a pulmonary sequestration. (**C**-**D**) Chest CT angiography shows (**C**) Increased caliber of the main pulmonary artery (29 mm) and (**D**) heterogeneous lung parenchyma density due to mosaic perfusion, both signs of pulmonary hypertension

**Fig. 4:**
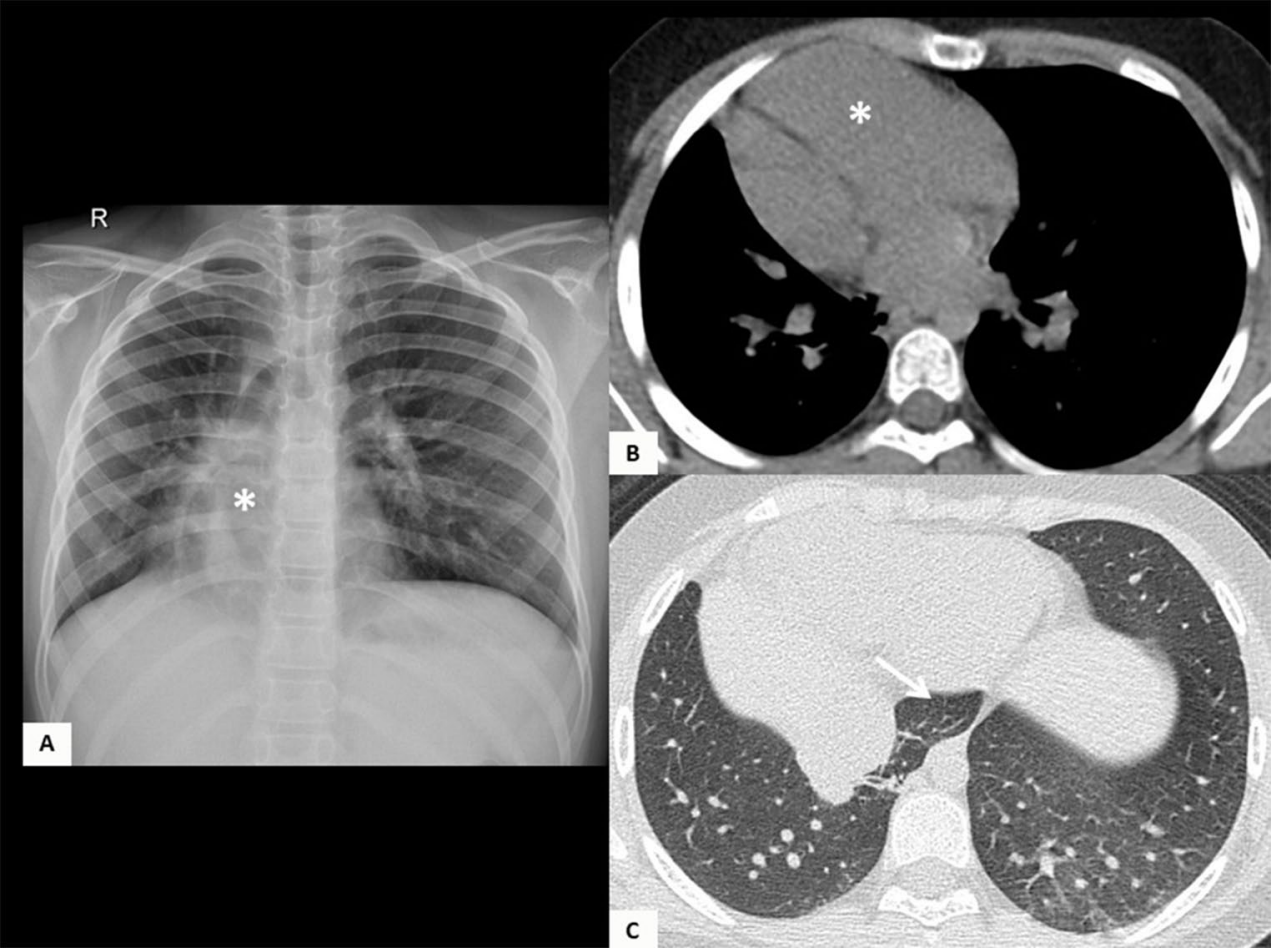
4-year-old female patient who had fallen a height, where findings were found incidentally. (**A**) Chest X-ray identified dextrocardia (asterisk). This was confirmed with a (**B**-**C**) chest CT scan revealing horseshoe lung (arrow in C)

Similarly, two patients (18.18%) had MRI. In the first case, findings in chest radiography were inconclusive, prompting the need for an MRI. In the second case, the patient provided MRI images and findings from an external institution. Both patients exhibited evidence of the Scimitar vein on the right side. One (9.09%) patient had suprahepatic Scimitar vein drainage, and another (9.09%) had infrahepatic Scimitar vein drainage. One of the patients had evidence of pulmonary hypoplasia, while the other patient had right pulmonary artery hypoplasia and right-sided bronchopulmonary sequestration. None of the patients who underwent MRI showed associated cardiac defects, horseshoe lung, cardiac dextroposition nor aortopulmonary collateral arteries (Table 2).

Among the eleven patients included in our study, echocardiography was performed on three individuals (27.27%), with findings consistent with Scimitar syndrome identified in two cases (18.18%). Importantly, two patients had undergone prior correction of ventricular septal defects. Of these two patients, one exhibited signs of the Scimitar anomaly on echocardiography, while the other did not. Notably, one of the patients who displayed evidence of the Scimitar syndrome on echocardiography had the anomaly left untreated, revealing drainage of the right inferior pulmonary vein into the inferior vena cava. All three patients showed dextroposition of the heart.

Five patients (45.45%) underwent cardiac catheterization to rule out pulmonary hypertension, determine the need for collateral embolization, and detect pulmonary sequestration. Among these patients, all five were found to have a left-to-right shunt, with QP/QS values of 1.16, 1.38, and 1.9. It is important to note that the QP/QS values for two patients were described only as very high without specific numerical values. Regarding median pulmonary pressure, recorded values were 41 mmHg, 19 mmHg, and 14 mmHg. One more patient was described as having elevated pressures without quantifiable records. However, all patients were described to have normal pulmonary vascular resistances, thus indicating pulmonary hyperperfusion, and justifying the decision to perform shunt closure.

Seven (63.64%) patients required surgical management, with the majority (45.45%) undergoing a single surgical stage. In three patients, correction of the Scimitar vein was performed along with the correction of other congenital heart defects, while the decision to operate on the other four depended on their hemodynamic stability (Table 3). Among these patients who underwent corrective procedures, the average hospital stay was 24.14 days (IQR: 19–29 days), with an average intensive care unit stay of 8.8 days (IQR: 3–16 days). The average duration of intubation was 5.83 h (IQR: 4.5–8 h). Conversely, patients who did not undergo surgery only required outpatient medication adjustments. Two patients died due to unrelated complications. One of the patients concurrently had extensive necrotizing enterocolitis, which, combined with episodes of severe hypoglycemia and malnutrition, contributed to the patient entering cardiorespiratory arrest from which it did not recover. The second patient had herniation of the middle lobe of the right lung with compression of the left lobe. After discussion in a medical meeting, it was considered appropriate to perform the left lower and middle lobectomy. The patient underwent surgery, and during the procedure, the patient experienced profuse bleeding and cardiac arrest, from which resuscitation efforts were unsuccessful.

**Table 3:** Treatment and clinical follow-up characteristics of patients

		*n* = 11 (%)
Required surgical management	7 (63,64%)	
Surgical stages	1 stage	5 (45,45%)
	2 stages	2 (18,18%)
RACHS-2 clasification	RACHS 1	4 (36,36%)
	RACHS 2	5 (45,45%)
	RACHS 3	2 (18,18%)
Scimitar vein correction	6 (54,55%)	
Ligation/embolization of the vascular supply to the sequestrated lobe	5 (45,45%)	
Mortality after surgery	2 (18,18%)	
Follow Up (Years)	3,64 (0–8)	

## Discussion

Scimitar syndrome is a rare congenital condition, which can go unnoticed or significantly impact a patient’s quality of life. Therefore, its incidence is considered low, as many patients can be asymptomatic. The probability of developing symptoms after diagnosis is 1% per year [11]. Three possible forms have been suggested: an infantile form, in which the patient is symptomatic and presents with pulmonary hypertension; an adult form characterized by the absence of symptoms during childhood; and a congenital form associated with other heart malformations [7, 9]. However, some authors consider only the first two. In our case we take into consideration all, and the predominant observation in our study was the prevalence of the infantile form, with no reported instances of adult manifestation, being the oldest patient seventeen years of age. Considering that our analysis only included patients who visited our institution during a lapse of eleven years, a significant number of cases were reported. Furthermore, although the literature indicates a 2:1 ratio with a predominance of females [12], in this scenario, a nearly equal frequency was observed, with a slightly higher frequency of females compared to the opposite sex.

As a result of this malformation, there is an alteration in the relationship between pulmonary flow and systemic flow due to the presence of a left-to-right shunt as observed in five of our patients [11, 12]. The occurrence of symptoms and their severity will depend on various factors, such as the number of pulmonary veins connecting to the scimitar vein, the pulmonary lobes or segments from which the anomalous vein originates, and the differences in resistance between the normally functioning pulmonary vascular areas and the anomalous ones [12]. Therefore, when scimitar syndrome affects at an early stage, growth disturbances, tachypnea, cyanosis, heart failure, recurrent lung infections, and bronchiectasis can be observed, resulting in higher mortality at a younger age [10, 13]. Contrastingly, in adulthood, there is a higher frequency of electrocardiographic abnormalities, with right bundle branch block being the most common, associated with right heart volume overload [10, 13]. It must be noted, although more studies are pending, adults tend to present a mild form of the syndrome, while infantile form have a higher incidence of symptoms, extracardiac anomalies and pulmonary hypertension, among others [14].

In the present study, approximately 80% of the patients experienced symptoms, with cyanosis being the most common manifestation, and likewise, the presence of recurrent pulmonary infections was reported in a significant number of patients. As no adult form was identified within our study cohort, it is not feasible to draw conclusions regarding variations in symptomatic presentation across different forms, and consequently, no disparities in symptom severity can be inferred. Nevertheless, the presence of respiratory insufficiency, dextroposition of the heart, and hypoplasia of the right lung, constitute the triad of findings should alert clinicians to this syndrome regardless of age [10, 15]. In our study cohort, approximately 73% of patients exhibited varying degrees of respiratory insufficiency as indicated by alterations on the NYHA scale. Additionally, six patients displayed dextroposition of the heart, detectable through at least one diagnostic imaging modality employed, while approximately half of the patients reported hypoplasia of the right lung, therefore proving that the triad can be found in the majority of the patients with scimitar syndrome.

While there is currently no established genetic basis for this syndrome, there have been suggestions that genes *STRC* and *CATSPER2* may play a role in its development [16]. Within the observed group, genetic patterns were not studied thoroughly, although no relevant family or personal histories were noted.

As previously mentioned, the diagnosis of this syndrome relies on a clinical component supported by imaging. For instance, chest X-rays often reveal suggestive features of the syndrome [17, 18]. Some studies indicate that the scimitar sign is the most common finding, occurring in up to 70% of chest X-rays [18], while other studies show right ventricular overgrowth as the primary finding. In our context, the scimitar sign was observed in approximately 82% of cases. Moreover, echocardiography serves as well to detect anomalous drainage and helps to identify concomitant cardiac defects [9]. However, transthoracic technique has a diagnostic limitation in up to 33% of the cases, and overall offers a small field of view, a variable acoustic window, and difficulty in delineating extracardiac vascular structures in their entirety [19, 20]. In our group this diagnostic image was not required in most of the patients. On the other hand, chest CT, whether 3D or not, or MRI, reliably establish cardiac anatomy and visualization of extracardiac arterial and venous anatomy [9, 20]. Therefore, if surgical intervention is necessary and previous images insufficiently depict cardiac morphology, these images could be used. The decision of whether to use chest CT, 3D or not, or MRI, should be based on institutional equipment availability, scheduling and urgency of the image, and the patients ability to cooperate [20]. In contrast to findings in other studies [17], our observed group’s chest CT scans showed a higher frequency of suprahepatic drainage than infrahepatic drainage, with a similar frequency of pulmonary hypoplasia in both. Furthermore, cardiac catheterization and angiography confirm diagnosis and offers important hemodynamic data such as shunt direction, assess for pulmonary hypertension, identify pulmonary vascular resistances, and enable surgical interventions when needed, among others [9].

Considering that there are no guidelines establishing criteria for the use of one image over another, the selection of these depends on the individual conditions of each patient and the clinical suspicions of the physician. Likewise, due to the low frequency of this syndrome, there is heterogeneity in the results when evaluating imaging findings [17]. Within our cohort, the most common diagnostic images were chest X-ray and CT scan, followed by catheterization, in cases where pulmonary hypertension, presence of pulmonary collaterals, or pulmonary sequestration was suspected.

Furthermore, the treatment approach hinges on both the severity of the syndrome and the clinical manifestations displayed by the patient. Consequently, the mode of presentation assumes a critical role in determining the optimal course of action, given the ongoing debate regarding the appropriate timing for surgical intervention. For infantile forms and those associated with other congenital heart diseases in the vast majority of cases, surgical intervention is required at some point in their life, without underestimating the population that can be managed exclusively with medical treatment [21]. However, patients with other congenital heart malformations have a much higher risk of death if defects are not corrected early [21]. On our case, three out of the seven patients driven into scimitar vein correction had at least one other congenital heart defect, being the most common the ventricular septal defect, present in the three patients. The other four patients who were intervened, the decision was made according to their hemodynamic repercussions, where unstable patients were taken into surgery. It is noteworthy that among the patients who underwent surgery, five exhibited cyanosis, two presented with pulmonary hypertension and six were classified as NYHA class II or higher. None of the medically treated presented the previously mentioned. Factors such as the presence or absence of shunts, pulmonary blood flow, the number of anomalous veins, the presence of concomitant heart or lung disease, and severity of symptoms are some of the variables that may be considered when deciding whether to perform a surgical procedure [22, 23].

While most surgical interventions aim to redirect pulmonary venous blood to the left atrium, there is currently no established consensus on the approach and indications for surgery [21–23]. As a result, the decision largely relies on the surgeon’s preference and expertise. The surgical approach, whether it involves intra-atrial baffle or Scimitar vein reimplantation, is primarily determined by anatomical and pathological characteristics [23]. In instances where the intra-atrial baffle technique is employed, a pericardium patch may be utilized, if necessary, to prevent obstruction of inferior vena cava (IVC) return, which [23]. New multipatch techniques have been developed and proven to be effective and safe in the short and intermediate term follow ups [24]. Further studies should be made in order to comprehend its effects on long term follow ups [24]. In our case, the intra-atrial baffle was the preferred method, in which a rerouting of the anomalous pulmonary vein flow to the left atrium is made through the formation of a tunnel using an intra-atrial patch [9, 24]. A single patient necessitated pneumonectomy due to the inability to correct the anomalous drainage of the scimitar vein. Additionally, this patient underwent multiple surgical interventions afterwards to address the need for replacement of the lung expander, attributable to growth-related challenges. The selection of patients who were taken into surgery was determined by the presence of additional congenital heart defects requiring surgical intervention or the presence of hemodynamic repercussion.

Regarding complications after surgical procedure, the most common following Scimitar vein correction is the occurrence of pulmonary vein obstruction, a condition that may affect up to half of patients postoperatively with no significant difference in complication rate regarding procedure [24]. Hence, there exists controversy regarding the potential efficacy of these procedures in long-term symptom alleviation for the patient. On our group no patient reported pulmonary vein obstruction or any other complications on their follow-ups. It is important to delineate that follow-up in this context was considered as any instance of a subsequent ambulatory visit or hospital admission to our institution after the diagnosis of scimitar syndrome or surgical intervention, specifically evaluating the presence of symptoms or hospitalization due to any associated complication. Aside from the previously mentioned patient who required further surgical procedures, only two patients more presented new hospitalizations due to sickle cell crisis and Dengue fever. However, considering the retrospective nature and single center based of our study, the possibility that they may have sought consultation at other centers is not ruled out.

On the contrary, medical therapy is typically employed to address symptoms related to pulmonary hypertension and heart failure, or considered for patients for whom surgery is not a viable option or those experiencing recurrent pulmonary infections [11, 23]. Diuretics constitute the primary pharmacological intervention employed for treatment. The primary objectives of medical treatment include enhancing respiratory function, alleviating lung congestion, and lowering pulmonary resistance [11, 23]. The adult form, patients often tolerate medical management very well, though the mortality of patients who undergo surgical intervention is 0% [21]. Within our cohort, four patients were subjected to sole to medical therapy and proved to be effective, therefore not requiring surgical intervention throughout the observed study period. Further investigations into their follow-ups are imperative to provide a comprehensive assessment of the effectiveness and utility of medical treatment in these patients.

In terms of mortality, our study yields results comparable to those of multicenter studies conducted globally [7, 25]. Although the age at the first intervention differed from what was observed in our cohort, a small number of deaths were reported overall. In contrast, the causes of death in our group are unrelated to the procedures performed or to cardiopulmonary affections, contrary to what is observed in the previously mentioned studies [7, 25]. The average follow-up time in our study was 3,64 years, during which nine patients remained free of symptoms related to scimitar vein and did not require additional procedures, further highlighting a contrast with other studies where reinterventions were necessary. As mentioned earlier, only one patient required pneumonectomy and further surgical interventions to change pulmonary expander without further need of different procedures. Although our study’s sample size is limited compared to broader international investigations, it represents the first comprehensive evaluation of this condition in Latin America, encompassing clinical and imaging observations, as well as surgical and medical outcomes. Notably, our study boasts the largest cohort group in the region, where previously only case reports have been documented. It is important to acknowledge several limitations in our study, including single institution, retrospective review, small sample size, and absence of strict follow-up. While it remains premature to assess the long-term progression, an anticipated improvement in life expectancy is expected for our patients.

## Conclusion

The scimitar syndrome remains a condition with many yet-to-be-revealed aspects regarding diagnostic tools usage and criteria for medical or surgical treatments. These patients require a multidisciplinary approach along with rapid and timely care as soon as symptoms begin to emerge, ultimately affecting the functionality and quality of life of both the patient and their families. Chest radiography and CT scan proved to be the most used and effective tools for its diagnostic. Surgical intervention when concomitant with other congenital heart defects or when hemodynamic repercussion proved to be sufficient criteria for a favorable surgical outcome. Medical treatment for patients with mild to moderate symptoms proved to be effective on their follow-ups. While it is still too early to assess the long-term evolution of our patients considering our study’s limitations, an improvement in their life expectancy is anticipated.

## Data Availability

No datasets were generated or analysed during the current study.
